# Modular strategies for spatial mapping of diverse cell type data of the mouse brain

**DOI:** 10.21203/rs.3.rs-6289741/v1

**Published:** 2025-04-09

**Authors:** Nicholas J. Tustison, Min Chen, Fae N. Kronman, Jeffrey T. Duda, Clare Gamlin, Mia G. Tustison, Michael Kunst, Rachel Dalley, Staci Sorenson, Quanxin Wang, Lydia Ng, Yongsoo Kim, James C. Gee

**Affiliations:** 1 Department of Radiology and Medical Imaging, University of Virginia, Charlottesville, VA; 2 Department of Radiology, University of Pennsylvania, Philadelphia, PA; 3 Department of Neural and Behavioral Sciences, Penn State University, Hershey, PA; 4 Allen Institute for Brain Science, Seattle, WA

## Abstract

Large-scale, international collaborative efforts by members of the BRAIN Initiative Cell Census Network (BICCN) consortium are aggregating the most comprehensive reference database to date for diverse cell type profiling of the mouse brain, which encompasses over 40 different multi-modal profiling techniques from more than 30 research groups. One central challenge for this integrative effort has been the need to map these unique datasets into common reference spaces such that the spatial, structural, and functional information from different cell types can be jointly analyzed. However, significant variation in the acquisition, tissue processing, and imaging techniques across data types makes mapping such diverse data a multifarious problem. Different data types exhibit unique tissue distortion and signal characteristics that precludes a single mapping strategy from being generally applicable across all cell type data. Tailored mapping approaches are often needed to address the unique barriers present in each modality. This work highlights modular atlas mapping strategies developed across separate BICCN studies using the Advanced Normalization Tools Ecosystem (ANTsX) to map spatial transcriptomic (MERFISH) and high-resolution morphology (fMOST) mouse brain data into the Allen Common Coordinate Framework (AllenCCFv3), and developmental (MRI and LSFM) data into the Developmental Common Coordinate Framework (DevCCF). We discuss common mapping strategies that can be shared across modalities and driven by specific challenges from each data type. These mapping strategies include novel open-source contributions that are made publicly available through ANTSX. These include 1) a velocity flow-based approach for continuously mapping developmental trajectories such as that characterizing the DevCCF and 2) an automated framework for determining structural morphology solely through the leveraging of publicly resources. Finally, we provide general guidance to aid investigators to tailor these strategies to address unique data challenges without the need to develop additional specialized software.

## 1 Introduction

Over the past decade there have been significant advancements in mesoscopic single-cell analysis of the mouse brain. It is now possible to track single neurons in mouse brains^1^, observe whole brain developmental changes on a cellular level^2^, associate brain regions and tissues with their genetic composition^3^, and locally characterize neural connectivity^4^. Much of these scientific achievements have been made possible due to breakthroughs in high resolution cell profiling and imaging techniques that permit submicron, multi-modal, 3-D characterizations of whole mouse brains. Among these include advanced techniques such as micro-optical sectioning tomography^6^, tissue clearing^1,7^, spatial transcriptomics^9^, and single-cell genomic profiling^10^, which have greatly expanded the resolution and specificity of single-cell measurements in the brain.

Recent efforts by the National Institutes of Health’s Brain Research Through Advancing Innovative Neurotechnologies (BRAIN) Initiative has pushed for large-scale, international collaborative efforts to utilize these advanced single-cell techniques to create a comprehensive reference database for high-resolution transcriptomic, epigenomic, structural and imaging data of the mouse brain. This consortium of laboratories and data centers, known as the BRAIN Initiative Cell Census Network (BICCN), has archived datasets encompassing over 40 different multi-modal profiling techniques from more than 30 research groups, each providing unique characterizations of distinct cell types in the brain^11^. Several of these modalities have been further developed into reference atlases to facilitate spatial alignment of individual brains and different data types into a common coordinate framework (CCF), thus allowing diverse single-cell information to be analyzed in an integrated manner. The most notable of these atlases is the Allen Mouse Brain Common Coordinate Framework (AllenCCFv3)^12^, which serves as a primary target coordinate space for much of the work associated with the BICCN. Other atlases include modality-specific atlases^13–15^, and spatiotemporal atlases^16,17^ for the developing mouse brain.

### 1.1 Mouse brain mapping

The cross-modality associations that can be learned from mapping different cell type data into a CCF is critical for improving our understanding of the complex relationships between cellular structure, morphology, and genetics in the brain. However, finding an accurate mapping between each individual mouse brain and a CCF is a challenging and heterogeneous task. There is significant variance in the imaging protocols across different cell type data as well as different tissue processing and imaging methods which can potentially introduce tissue distortion and signal differences^18,19^. Certain modalities can have poor intensity correspondence with the CCF, negatively impacting image alignment accuracy. Studies targeting specific regions or cell types can lead to missing anatomical correspondences. Other considerations include artifacts such as tissue distortion, holes, bubbles, folding, tears, and missing sections in the data that often require manual correction^20–23^. Given the diversity of these challenges, it is unlikely any single mapping approach can be generally applicable across all cell type data. Diverse, and often specialized, strategies are needed to address the unique barriers present for mapping each modality.

Existing solutions to address mapping cell type data into the AllenCCFv3 falls broadly into three main categories. The first consists of integrated processing platforms that directly provide mapped data to the users. These include the Allen Brain Cell Atlas^24^ for the Allen Reference Atlas (ARA) and associated data, the Brain Architecture Portal^25^ for combined ex vivo radiology and histology data, OpenBrainMap^26^ for connectivity data, and the Image and Multi-Morphology Pipeline^27^ for high resolution morphology data. These platforms provide users online access to pre-processed, multi-modal cell type data that are already mapped to the AllenCCFv3. The platforms are designed such that the data is interactively manipulated by users through integrated visualization software that allow users to spatially manipulate and explore each dataset within the mapped space. While highly convenient for investigators who are interested in studying the specific modalities provided by these platforms, these systems can be limited in flexibility, general applicability, and public availability. As a result, investigators often find it difficult to apply the same mapping solutions to their own data.

The second category comprises specialized approaches specifically designed for mapping one or more modalities into a CCF. These approaches use combinations of specialized manual and automated processes that address specific challenges in each modality. Examples include approaches for mapping histology^28–30^, magnetic resonance imaging (MRI)^37^, micro-computed tomography (microCT)^35,37^, light-sheet fluorescence microscopy (LSFM)^34,36–39^, fluorescence micro-optical sectioning tomography (fMOST)^15,40^ and transcriptomic data^41–43^. As specialized approaches, these techniques tend to boast higher mapping accuracy, robustness, and ease of use. Conversely, their specialized designs often rely on base assumptions regarding the data type that can make them rigid and difficult to adapt for new modalities or unexpected artifacts and distortions in the data. Adapting these specialize software tools to use with new data can require significant development, validation time, and engineering expertise that may not be readily available for all investigators.

The last category consists of modular mapping approaches constructed using general image analysis toolkits, which are software packages that include modular image processing, segmentation and registration tools that have been previously developed, and validated for multiple application areas. Examples of such toolkits include elastix^44^, Slicer3D^45^, ANTsX^46^, and several others which have all been applied towards mouse brain spatial mapping. The main challenge, in these mouse-specific study scenarios, is that tailored pipelines often need be constructed from available software components. Investigators must therefore be familiar with the these tools for formulating new or adapting existing pipelines. However, in comparison to previously described specialized mapping approaches, these approaches are often easier to create and prone to robustness, being typically constructed from pipeline components which have been previously vetted in other contexts. In this work, we highlight such mapping strategies designed using the ANTsX framework to map distinct mouse cell type data with different characteristics into existing CCFs.

### 1.2 Advanced Normalization Tools (ANTsX)

The Advanced Normalization Tools Ecosystem (ANTsX) has been used in a number of applications for mapping mouse brain data as part of core processing steps in various workflows^30,47–50^, particularly its pairwise, intensity-based image registration capabilities^51^ and bias field correction^52^. Historically, ANTsX development is originally based on fundamental approaches to image mapping^53–55^, particularly in the human brain, which has resulted in core contributions to the field such as the widely-used Symmetric Normalization (SyN) algorithm^51^. Since its development, various independent platforms have been used to evaluate ANTsX image registration capabilities in the context of different application foci which include multi-site brain MRI data^56^, pulmonary CT data^57^, and most recently, multi-modal brain registration in the presence of tumors^58^.

Apart from its registration capabilities, ANTsX comprises additional functionality such as template generation^59^, intensity-based segmentation^60^, preprocessing^52,61^, deep learning networks^46^, and other utilities relevant to brain mapping (see Table 1). The use of the toolkit has demonstrated high performance in multiple application areas (e.g., consensus labeling^62^, brain tumor segmentation^63^, and cardiac motion estimation^64^). Importantly, ANTsX is built on the Insight Toolkit (ITK)^65^ deriving benefit from the open-source community of scientists and programmers as well as providing an important resource for algorithmic development, evaluation, and improvement.

With respect to mouse cell type data, ANTsX provides a comprehensive toolset which serves as a basis for developing modular frameworks for mapping diverse image data into common coordinate frameworks (CCFs). Herein, we highlight its application for mapping data from separate BICCN projects focused on distinct data types: morphology data using fluorescence micro-optical sectioning tomography (fMOST), spatial transcriptomics from multiplexed error-robust fluorescence in situ hybridization (MERFISH) data, and time-series developmental data using light sheet fluorescence microscopy (LSFM) and magnetic resonance imaging (MRI). We describe both shared and targeted strategies developed to address the specific challenges of these modalities.

### 1.3 Novel ANTsX-based open-source contributions

We introduce two novel inclusions to the ANTsX toolset that were developed as part of the MRI mapping and analysis pipeline for the Developmental Common Coordinate Framework (DevCCF). Consistent with previous ANTsX development, newly introduced capabilities introduced below are available through ANTsX (specifically, via R and Python ANTsX packages), and illustrated through self-contained examples in the ANTsX tutorial (https://tinyurl.com/antsxtutorial) with a dedicated GitHub repository specific to this work (https://github.com/ntustison/ANTsXMouseBrainMapping). To complement standard preprocessing steps (e.g., bias correction, brain masking), additional mouse brain specific tools have also been introduced to the ANTsX ecosystem, such as section reconstruction and landmark-based alignment with corresponding processing scripts (https://github.com/dontminchenit/CCFAlignmentToolkit).

#### 1.3.1 Continuously mapping the DevCCF developmental trajectory with a velocity flow model

Recently, the Developmental Common Coordinate Framework (DevCCF) was introduced to the mouse brain research community as a public resource^16^ comprising symmetric atlases of multi-modal image data and anatomical segmentations defined by developmental ontology. These templates sample the mouse embryonic days E11.5, E13.5, E15.5, E18.5 and postnatal days P4, P14, and P56. Modalities include LSFM and at least four MRI contrasts per developmental stage. Anatomical parcellations are also available for each time point and were generated from ANTsX-based mappings of gene expression and other cell type data. Additionally, the P56 template was integrated with the AllenCCFv3 to further enhance the practical utility of the DevCCF. These processes, specifically template generation and multi-modal image mapping, were performed using ANTsX functionality in the presence of image mapping difficulties such as missing data and tissue distortion.

Given the temporal gaps in the discrete set of developmental atlases, we also provide an open-source framework for inferring correspondence within the temporally continuous domain sampled by the existing set of embryonic and postnatal atlases of the DevCCF. This recently developed functionality permits the generation of a diffeomorphic velocity flow transformation model^66^, influenced by previous work^67^. The resulting time-parameterized velocity field spans the stages of the DevCCF where mappings between any two continuous time points within the span bounded by the E11.5 and P56 atlases are determined by numerical integration of the optimized velocity field.

#### 1.3.2 Automated structural parcellations of the mouse brain

In contrast to the pipeline development in human data^46^, limited tools exist yet to create adequate training data for automated parcellations of the mouse brain. In addition, mouse brain data acquisition often has unique issues, such as lower data quality or sampling anisotropy which limits its applicability to high resolution resources (e.g., AllenCCFv3, DevCCF), specifically with respect to the corresponding granular brain parcellations derived from numerous hours of expert annotation leveraging multi-modal imaging resources.

Herein, we introduce a mouse brain parcellation pipeline for multi-modal MRI comprising two novel deep learning components: two-shot learning brain extraction from data augmentation of two ANTsX templates generated from two open datasets^68,69^ and single-shot brain parcellation derived from the AllenCCFv3 labelings mapped to the corresponding DevCCF P56 template. Although we anticipate that this pipeline will be beneficial to the research community, this work demonstrates more generally how one can leverage ANTsX tools and other public resources for developing quantitative mouse brain morphological tools. Evaluation is performed on an independent open dataset^70^ comprising longitudinal acquisitions of multiple specimens.

## 2 Results

### 2.1 AllenCCFv3 brain image mapping

#### 2.1.1 Mapping multiplexed error-robust fluorescence in situ hybridization (MERFISH) data

##### Overview.

**Figure 1:**
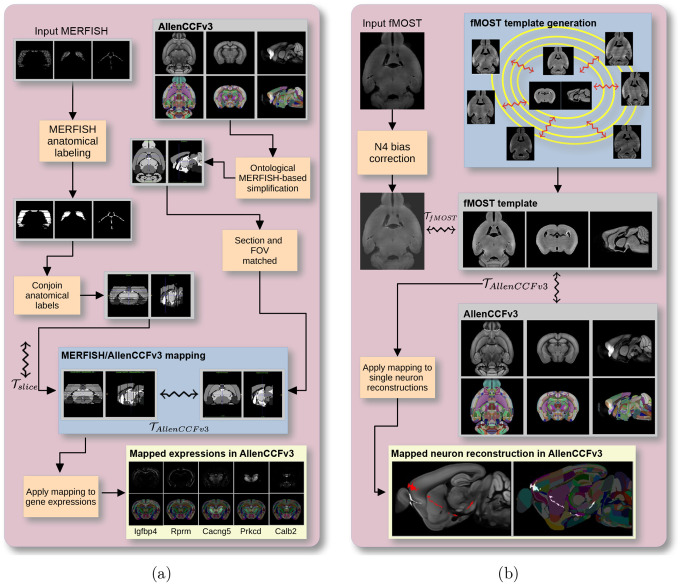
Diagram of the two ANTsX-based pipelines for mapping (a) MERFISH and (b)fMOST data into the space of AllenCCFv3. Each generates the requisite transforms, 𝒯, to map individual images to the CCF.

The ANTsX framework was used to develop a pipeline for mapping multiplexed error-robust fluorescence in situ hybridization (MERFISH) spatial transcriptomic mouse data onto the AllenCCFv3 (see Figure 1(a)). This pipeline, used recently in creating a high-resolution transcriptomic atlas of the mouse brain^50^, performs mappings by first generating anatomical labels from tissue related gene expressions in the MERFISH data, and then spatially matching these labels to corresponding anatomical tissue parcellations in the AllenCCFv3. The pipeline consists of MERFISH data specific preprocessing which includes section reconstruction, mapping corresponding anatomical labels between AllenCCFv3 and the spatial transcriptomic maps of the MERFISH data, and matching MERFISH sections to the atlas space. Following preprocessing, two main alignment steps were performed: 1) 3-D global affine mapping and section matching of the AllenCCFv3 into the MERFISH data and 2) 2-D global and deformable mapping between each MERFISH section and matched AllenCCFv3 section. Mappings learned via each step in the pipeline are preserved and concatenated to provide point-to-point correspondence between the original MERFISH data and AllenCCFv3, thus allowing individual gene expressions to be transferred into the AllenCCFv3.

##### Data.

MERFISH mouse brain data was acquired using a previously detailed procedure^50^. Briefly, a brain of C57BL/6 mouse was dissected according to standard procedures and placed into an optimal cutting temperature (OCT) compound (Sakura FineTek 4583) in which it was stored at −80°C. The fresh frozen brain was sectioned at 10*μm* on Leica 3050 S cryostats at intervals of 200*μm* to evenly cover the brain. A set of 500 genes were imaged that had been carefully chosen to distinguish the ~5200 clusters of our existing RNAseq taxonomy. For staining the tissue with MERFISH probes, a modified version of instructions provided by the manufacturer was used^50^. Raw MERSCOPE data were decoded using Vizgen software (v231). Cells were segmented based on DAPI and PolyT staining using Cellpose^71,72^. Segmentation was performed on a median z-plane (fourth out of seven) and cell borders were propagated to z-planes above and below. To assign cluster identity to each cell in the MERFISH dataset, we mapped the MERFISH cells to the scRNA-seq reference taxonomy.

##### Evaluation.

Alignment of the MERFISH data into the AllenCCFv3 was qualitatively assessed by an expert anatomist at each iteration of the registration using known correspondence of gene markers and their associations with the AllenCCFv3. As previously reported^50^, further assessment of the alignment showed that, of the 554 terminal regions (gray matter only) in the AllenCCFv3, only seven small subregions were missed from the MERFISH dataset: frontal pole, layer 1 (FRP1), FRP2/3, FRP5; accessory olfactory bulb, glomerular layer (AOBgl); accessory olfactory bulb, granular layer (AOBgr); accessory olfactory bulb, mitral layer (AOBmi); and accessory supraoptic group (ASO).

#### 2.1.2 Mapping fluorescence micro-optical sectioning tomography (fMOST) data

##### Overview.

We developed a pipeline for mapping fluorescence micro-optical sectioning tomography (fMOST) mouse brain images into the AllenCCFv3 (see Figure 1(b)). The pipeline is adapted from previously developed frameworks for human brain mapping^59^, and uses a modality specific (fMOST) average atlas to assist in the image registration and mapping. This approach has been well validated in human studies^73–75^, and successfully used in other mouse data^12,15,34^. Briefly, we construct an intensity- and shape-based average fMOST atlas using 30 fMOST images to serve as an intermediate registration target for mapping fMOST images from individual specimens into the AllenCCFv3. Preprocessing steps include downsampling to match the 25*μm* isotropic AllenCCFv3, acquisition-based stripe artifact removal, and inhomogeneity correction^52^. Preprocessing also includes a single annotation-driven registration to establish a canonical mapping between the fMOST atlas and the AllenCCFv3. This step allows us to align expert determined landmarks to accurately map structures with large morphological differences between the modalities, which are difficult to address using standard approaches. Once this canonical mapping is established, standard intensity-based registration is used to align each new fMOST image to the fMOST specific atlas. This mapping is concatenated with the canonical fMOST atlas-to-AllenCCFv3 mapping to further map each individual brain into the latter without the need to generate additional landmarks. Transformations learned through this mapping can be applied to single neuron reconstructions from the fMOST images to evaluate neuronal distributions across different specimens into the AllenCCFv3 for the purpose of cell census analyses.

##### Data.

The high-throughput and high-resolution fluorescence micro-optical sectioning tomography (fMOST)^76,77^ platform was used to image 55 mouse brains containing gene-defined neuron populations, with sparse transgenic expression^78,79^. In short, the fMOST imaging platform results in 3-D images with voxel sizes of 0.35 × 0.35 × 1.0*μm*^3^ and is a two-channel imaging system where the green channel displays the green fluorescent protein (GFP) labeled neuron morphology and the red channel is used to visualize the counterstained propidium iodide cytoarchitecture. The spatial normalizations described in this work were performed using the red channel, which offered higher tissue contrast for alignment, although other approaches are possible including multi-channel registration.

##### Evaluation.

Evaluation of the canonical fMOST atlas to Allen CCFv3 mapping was performed via quantitative comparison at each step of the registration and qualitative assessment of structural correspondence after alignment by an expert anatomist. Dice values were generated for the following structures: whole brain, 0.99; fimbria, 0.91; habenular commissure, 0.63; posterior choroid plexus, 0.93; anterior choroid plexus, 0.96; optic chiasm, 0.77; caudate putamen, 0.97. Similar qualitative assessment was performed for each fMOST specimen including the corresponding neuron reconstruction data.

### 2.2 Continuously mapping the DevCCF developmental trajectory with a velocity flow model

The DevCCF is an openly accessible resource for the mouse brain research community^80^. It consists of multi-modal MRI and LSFM symmetric ANTsX-generated templates^59^ sampling the mouse brain developmental trajectory, specifically the embryonic (E) and postnatal (P) days E11.5, E13.5, E15.5, E18.5 P4, P14, and P56. Each template space includes structural labels defined by a developmental ontology. Its utility is also enhanced by a coordinated construction with AllenCCFv3. Although this work represents a significant contribution, the gaps between time points potentially limit its applicability which could be addressed through the development of the ability to map not only between time points but also within and across time points.

To continuously generate transformations between the different stages of the DevCCF atlases, we developed a general velocity flow model approach which we apply to DevCCF-derived data. We also introduce this functionality into both the ANTsR and ANTsPy packages (for the latter, see ants.fit_time_varying_transform_to_point_sets(…)) for potential application to this and other analagous scenarios (e.g., modeling the cardiac and respiratory cycles). ANTsX, being built on top of ITK, uses an ITK image data structure for the 4-D velocity field where each voxel contains the *x*, *y*, *z* components of the field at that point.

#### 2.2.1 Data

**Figure 3:**
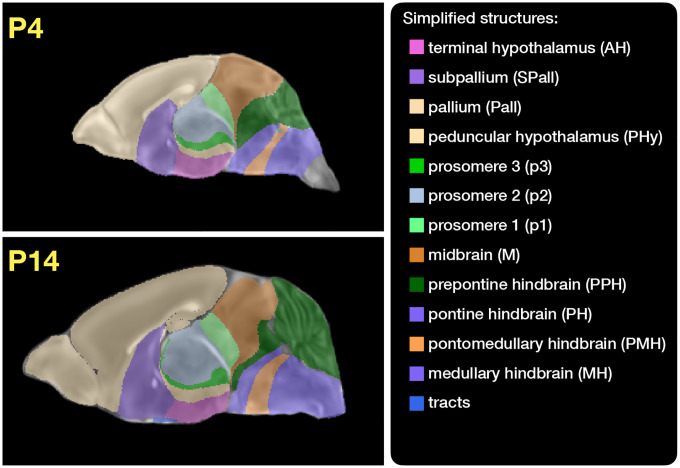
Annotated regions representing common labels across developmental stages which are illustrated for both P4 and P14.

Labeled annotations are available as part of the original DevCCF and reside in the space of each developmental template which range in resolution from 31.5 – 50*μ*m. Across all atlases, the total number of labeled regions exceeds 2500. From these labels, a common set of 26 labels (13 per hemisphere) across all atlases were used for optimization and evaluation. These simplified regions include: terminal hypothalamus, subpallium, pallium, peduncular hypothalamus, prosomere, prosomere, prosomere, midbrain, prepontine hindbrain, pontine hindbrain, pontomedullary hindbrain, medullary hindbrain, and tracts (see Figure 3).

Prior to velocity field optimization, all data were rigidly transformed to DevCCF P56 using the centroids of the common label sets. In order to determine the landmark correspondence across DevCCF stages, the multi-metric capabilities of ants.registration(…) were used. Instead of performing intensity-based pairwise registration directly on these multi-label images, each label was used to construct a separate fixed and moving image pair resulting in a multi-metric registration optimization scenario involving 24 binary image pairs (each label weighted equally) for optimizing diffeomorphic correspondence between neighboring time point atlases using the mean squares metric and the symmetric normalization transform^51^.

To generate the set of common point sets across all seven developmental atlases, the label boundaries and whole regions were sampled in the P56 atlas and then propagated to each atlas using the transformations derived from the pairwise registrations. We selected a sampling rate of 10% for the contour points and 1% for the regional points for a total number of points being per atlas being 173303 (*N*_*contour*_ = 98151 and *N*_*region*_ = 75152). Regional boundary points were weighted twice as those of non-boundary points during optimization.

#### 2.2.2 Velocity field optimization

The velocity field was optimized using the input composed of the seven corresponding point sets and their associated weight values, the selected number of integration points for the velocity field (*N* = 11), and the parameters defining the geometry of the spatial dimensions of the velocity field. Thus, the optimized velocity field described here is of size [256, 182, 360] (50*μ*m isotropic) ×11 integration points for a total compressed size of a little over 2 GB. This choice represented weighing the trade-off between tractability, portability, and accuracy. However, all data and code to reproduce the results described are available in the dedicated GitHub repository.

**Figure 4:**
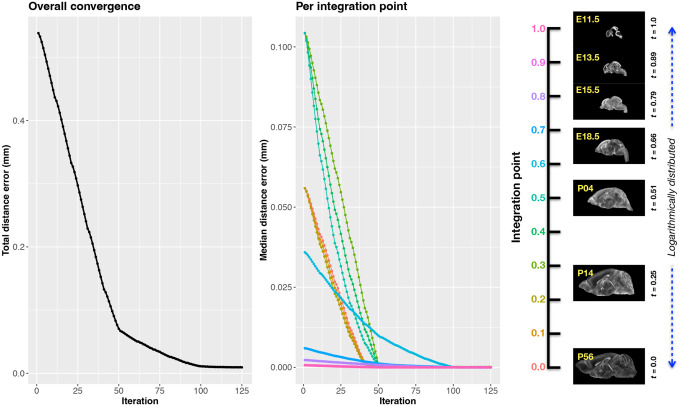
Convergence of the optimization of the velocity field for describing the transformation through the developmental stages from E11.5 through P56. Integration points in diagram on the right are color-coordinated with the center plot and placed in relation to the logarithmically situated temporal placement of the individual DevCCF atlases.

The normalized time point scalar value for each atlas/point-set in the temporal domains [0, 1] was also defined. Given the increasingly larger gaps in the postnatal time point sampling, we made two adjustments. Based on known mouse brain development, we used 28 days for the P56 data. We then computed the log transform of the adjusted set of time points prior to normalization between 0 and 1 (see the right side of Figure 4). This log transform, as part of the temporal normalization, significantly improves the temporal spacing of data.

The maximum number of iterations was set to 200 with each iteration taking approximately six minutes on a 2020 iMac (processor, 3.6 GHz 10-Core Intel Core i9; memory, 64 GB 2667 MHz DDR4) At each iteration we looped over the 11 integration points. At each integration point, the velocity field estimate was updated by warping the two immediately adjacent point sets to the integration time point and determining the regularized displacement field between the two warped point sets. As with any gradient-based descent algorithm, this field was multiplied by a small step size (*δ* = 0.2) before adding to the current velocity field. Convergence is determined by the average displacement error over each of the integration points. As can be seen in the left panel of Figure 4, convergence occurred around 125 iterations when the average displacement error over all integration points is minimized. The median displacement error at each of the integration points also trends towards zero but at different rates.

#### 2.2.3 The velocity flow transformation model

**Figure 5:**
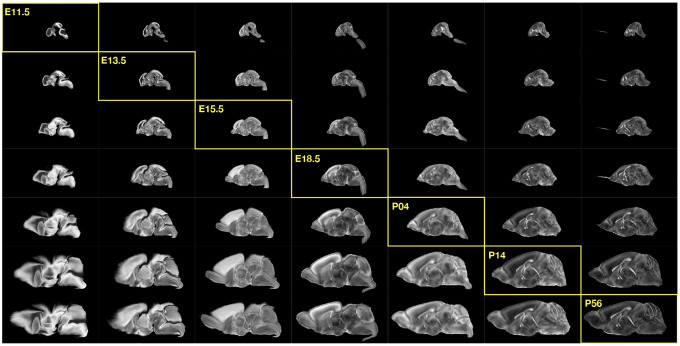
Mid-sagittal visualization of the effects of the transformation model in warping every developmental stage to the time point of every other developmental stage. The original images are located along the diagonal. Columns correspond to the warped original image whereas the rows represent the reference space to which each image is warped.

**Figure 6:**
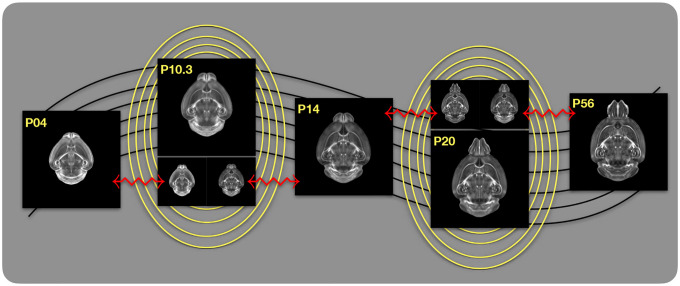
Illustration of the use of the velocity flow model for creating virtual templates at continuous time points not represented in one of the existing DevCCF time points. For example, FA templates at time point P10.3 and P20 can be generated by warping the existing temporally adjacent developmental templates to the target time point and using those images in the ANTsX template building process.

Once optimized, the resulting velocity field can be used to generate the deformable transform between any two continuous points within the time interval bounded by E11.5 and P56. As a demonstration, in Figure 5, we transform each atlas to the space of every other atlas using the DevCCF transform model. Additionally, one can use this transformation model to construct virtual templates in the temporal gaps of the DevCCF. Given an arbitrarily chosen time point within the normalized time point interval, the existing adjacent DevCCF atlases on either chronological side can be warped to the desired time point. A subsequent call to one of the ANTsX template building functions then permits the construction of the template at that time point. In Figure 6, we illustrate the use of the DevCCF velocity flow model for generating two such virtual templates for two arbitrary time points. Note that both of these usage examples can be found in the GitHub repository previously given.

### 2.3 Automated structural parcellations of the mouse brain

Brain parcellation strategies for the mouse brain are pivotal for understanding the complex organization and function of murine nervous system^81^. By dividing the brain into distinct regions based on anatomical, physiological, or functional characteristics, researchers can investigate specific areas in isolation and identify their roles in various behaviors and processes. For example, such parcellation schemes can help elucidate the spatial distribution of gene expression patterns^82^ as well as identify functional regions involved in specific cognitive tasks^83^.

**Figure 7:**
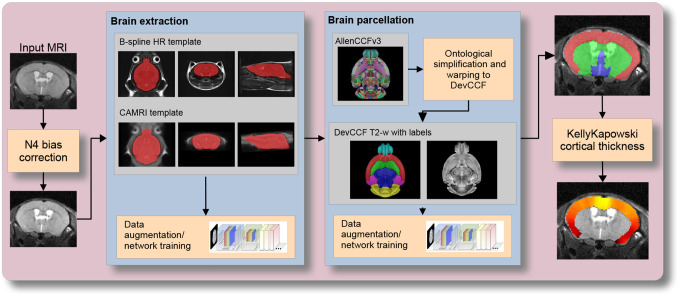
The mouse brain cortical parcellation pipeline integrating two deep learning components for brain extraction and brain parcellation prior to estimating cortical labels. Both deep learning networks rely heavily on aggressive data augmentation on templates built from open data and provide an outline for further refinement and creating alternative parcellations for tailored research objectives. Possible applications include voxelwise cortical thickness measurements.

Although deep learning techniques have been used to develop useful parcellation tools for human brain research (e.g., SynthSeg^84^, ANTsXNet^46^), analogous development for the mouse brain is limited. In addition, mouse data is often characterized by unique imaging issues such as extreme anisotropic sampling which are often in sharp contrast to the high resolution template-based resources available within the community, e.g., AllenCCFv3 and DevCCF. We demonstrate how one can use the ANTsX tools to develop a complete mouse brain structural morphology pipeline as illustrated in Figure 7 and detailed below.

#### 2.3.1 Few-shot mouse brain extraction network

In order to create a generalized mouse brain extraction network, we built whole-head templates from two publicly available datasets. The Center for Animal MRI (CAMRI) dataset^68^ from the University of North Carolina at Chapel Hill consists of 16 T2-w MRI volumes of voxel resolution 0.16×0.16×0.16*mm*^3^. The second high-resolution dataset^69^ comprises 88 specimens each with three spatially aligned canonical views with in-plane resolution of 0.08 × 0.08*mm*^2^ with a slice thickness of 0.5*mm*. These three orthogonal views were used to reconstruct a single high-resolution volume per subject using a B-spline fitting algorithm available in ANTsX^85^.

From these two datasets, two ANTsX templates^59^ were generated. Bias field simulation, intensity histogram warping, noise simulation, random translation and warping, and random anisotropic resampling in the three canonical directions were used for data augmentation in training an initial T2-w brain extraction network. This network was posted and the corresponding functionality was immediately made available within ANTsXNet, similar to our previous contributions to the community.

User interest led to a GitHub inquiry regarding possible study-specific improvements (https://github.com/ANTsX/ANTsPyNet/issues/133). This interaction led to the offering of a user-made third template and extracted brian mask generated from T2-w ex-vivo data with isotropic spacing of 0.08 mm in each voxel dimension. This third template, in conjunction with the other two, were used with the same aggressive data augmentation to refine the network weights which were subsequently posted and made available through ANTsPyNet using the function antspynet.mouse_brain_extraction(…).

#### 2.3.2 Single-shot mouse brain parcellation network

AllenCCFv3 and its hierarchical ontological labeling, along with the DevCCF, provides the necessary data for developing a tailored structural parcellation network for multi-modal imaging. The allensdk Python library permits the creation of any gross parcellation based on the AllenCCFv3 ontology. Specifically, using allensdk we coalesced the labels to the following six major structures: cerebral cortex, cerebral nuclei, brain stem, cerebellum, main olfactory bulb, and hippocampal formation. This labeling was mapped to the P56 component of the DevCCF for use with the T2-w template component.

The T2-w P56 DevCCF and labelings, in conjunction with the data augmentation described previously for brain extraction, were used to train the proposed brain parcellation network. This is available in ANTsXNet (e.g. in ANTsPyNet using antspynet.mouse_brain_parcellation(…)). Note that other brain parcellation networks have also been trained using alternative regions and parcellation schemes and are available in the same ANTsXNet functionality. One usage note is that the data augmentation used to train the network permits a learned interpolation in 0.08 mm isotropic space. Since the training data is isotropic and data augmentation includes downsampling in the canonical directions, each of the two networks learns mouse brain-specific interpolation such that one can perform prediction on thick-sliced images, as, for example, in these evaluation data, and return isotropic probability and thickness maps (a choice available to the user). This permits robust cortical thickness estimation even in the case of anisotropic data (see antspynet.mouse_cortical_thickness(…)).

#### 2.3.3 Evaluation

For evaluation, we used an additional publicly available dataset^70^ that is completely independent from the data used in training the brain extraction and parcellation networks. Data includes 12 specimens each imaged at seven time points (Day 0, Day 3, Week 1, Week 4, Week 8, Week 20) with in-house-generated brain masks for a total of 84 images. Spacing is anistropic with an in-plane resolution of 0.1 × 0.1*mm*^2^ and a slice thickness of 0.5*mm*.

**Figure 8:**
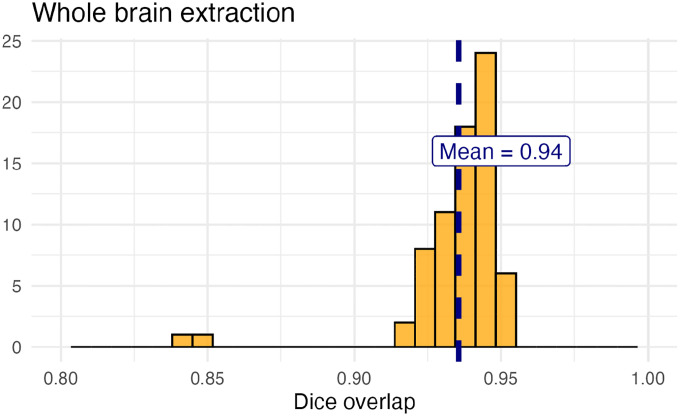
Evaluation of the ANTsX mouse brain extraction on an independent, publicly available dataset consisting of 12 specimens × 7 time points = 84 total images. Dice overlap comparisons with the user-generated brain masks provide good agreement with the automated results from the brain extraction network.

Figure 8 summarizes the whole brain overlap between the provided segmentations for all 84 images and the results of applying the proposed network. Also, since mapping to the AllenCCFv3 atlas is crucial for many mouse studies, we demonstrate the utility of the second network by leveraging the labeled regions to perform anatomically-explicit alignment using ANTsX multi-component registration instead of intensity-only registration. For these data, the whole brain extraction demonstrates excellent performance across the large age range. And although the intensity-only image registration provides adequate alignment, intensity with the regional parcellations significantly improves those measures.

## 3 Discussion

The diverse mouse brain cell type profiles gathered through BICCN and associated efforts provides a rich multi-modal resource to the research community. However, despite significant progress, optimized leveraging of these valuable resources is ongoing. A central component to data integration is accurately mapping novel cell type data into CCFs for subsequent processing and analysis. To meet these needs, tools for mapping mouse cell type data must be both generally accessible to a wide audience of investigators, and capable of handling distinct challenges unique to each data type. In this work, we described modular ANTsX-based pipelines developed to address the needs of three BICCN projects that cover distinct cell type data, including spatial transcriptomic, morphological, and developmental data. We highlighted how a modular toolbox like ANTsX can be tailored to address problems unique to each modality through leveraging a variety of ready-to-use powerful tools that have been previously validated in multiple application scenarios.

Our MERFISH pipeline provides an example of how to map high-resolution spatial transcriptomic data into the AllenCCFv3. While the techniques employed for mapping the sectioned data can be generally applicable to spatially transform other serial histology images, much of the pipeline was designed to specifically address known alignment challenges in the MERFISH data. Thus pipeline shows how general ANTsX tools can be adapted to target highly specialized problems in mouse cell type data.

In contrast to the MERFISH pipeline, our fMOST pipeline was designed to be a more general solution that can be employed in other modalities. The pipeline primarily uses previously developed ANTsX preprocessing and atlasing tools to map fMOST data into the AllenCCFv3. The key component of the pipeline is the use of a fMOST-specific average shape and intensity atlas to most effectively address image registration in this context. The mapping between the fMOST atlas is generated once and reused for each new fMOST image. Lastly, ANTsX provides point set transformation tools to allow the mappings found through the pipeline to be directly applied to associated single-cell reconstructions from the fMOST data to study neuronal morphology.

The pipeline for continuously mapping the DevCCF data is also available in ANTsX and is generally applicable for spatio-temporal mapping. With specific application to the DevCCF, despite the significant expansion of available developmental age templates beyond what existed previously, there are still temporal gaps in the DevCCF which can be potentially sampled by future research efforts. However, pioneering work involving time-varying diffeomorphic transformations allow us to continuously situate the existing templates within a velocity flow model. This allows one to determine the diffeomorphic transformation from any one temporal location to any other temporal location within the time span defined by the temporal limits of the DevCCF. This functionality is built on multiple ITK components including the B-spline scattered data approximation technique for field regularization and velocity field integration. This velocity field model permits intra-template comparison and the construction of virtual templates where a template can be estimated at any continuous time point within the temporal domain. This novel application can potentially enhance our understanding of intermediate developmental stages.

We also presented a mouse brain morphological pipeline for brain extraction and brain parcellation using single-shot and few-shot learning with aggressive data augmentation. This approach attempts to circumvent (or at least minimize) the typical requirement of large training datasets as with the human ANTsX pipeline analog. However, even given our initial success on independent data, we anticipate that refinements will be necessary. Given that the ANTsX toolkit is a dynamic effort undergoing continual improvement, we manually correct cases that fail and use them for future training and refinement of network weights as we have done for our human-based networks. And, as demonstrated, we welcome contributions from the community for improving these approaches which, generally, provide a way to bootstrap training data for manual refinement and future generation of more accurate deep learning networks in the absence of other applicable tools.

The ANTsX ecosystem is a powerful framework that has demonstrated applicability to diverse cell type data in the mouse brain. This is further evidenced by the many software packages that use various ANTsX components in their own mouse-specific workflows. The extensive functionality of ANTsX makes it possible to create complete processing pipelines without requiring the integration of multiple packages or lengthy software development. These open-source components not only perform well but are available across multiple platforms which facilitates the construction of tailored pipelines for individual study solutions. These components are also supported by years of development not only by the ANTsX development team but by the larger ITK community. Finally, as an extension to the BICCN program, ANTsX will be a powerful tool for the efforts of the BRAIN Initiative Cell Atlas Network (BICAN) to extend these efforts to the human brain.

## 4 Methods

The following methods are all available as part of the ANTsX ecosystem with analogous elements existing in both ANTsR (ANTs in R) and ANTsPy (ANTs in Python) with an ANTs/ITK C++ core. However, most of the development for the work described below was performed using ANTsPy. For equivalent calls in ANTsR, please see the ANTsX tutorial at https://tinyurl.com/antsxtutorial.

### 4.1 General ANTsX utilities

Although they focus on distinct data types, the three pipelines presented share common components that are generally applicable when mapping mouse cell type data. These include, addressing intensity biases and noise in the data, image registration to solve the mapping, creating custom templates and atlases from the data, and visualization of the results. Table 1 provides a brief summary of key general functionalities in ANTsX for addressing these challenges.

#### 4.1.1 Preprocessing: bias field correction and denoising

Bias field correction and image denoising are standard preprocessing steps in improving overall image quality in mouse brain images. The bias field, a gradual spatial intensity variation in images, can arise from various sources such as magnetic field inhomogeneity or acquisition artifacts, leading to distortions that can compromise the quality of brain images. Correcting for bias fields ensures a more uniform and consistent representation of brain structures, enabling more accurate quantitative analysis. Additionally, brain images are often susceptible to various forms of noise, which can obscure subtle features and affect the precision of measurements. Denoising techniques help mitigate the impact of noise, enhancing the signal-to-noise ratio and improving the overall image quality. The well-known N4 bias field correction algorithm^52^ has its origins in the ANTs toolkit which was implemented and introduced into the ITK toolkit, i.e. ants.n4_bias_field_correction(…). Similarly, ANTsX contains an implementation of a well-performing patch-based denoising technique^61^ and is also available as an image filter to the ITK community, ants.denoise_image(…).

#### 4.1.2 Image registration

The ANTs registration toolkit is a complex framework permitting highly tailored solutions to pairwise image registration scenarios^86^. It includes innovative transformation models for biological modeling^51,67^ and has proven capable of excellent performance^56,87^. Various parameter sets targeting specific applications have been packaged with the different ANTsX packages, specifically ANTs, ANTsPy, and ANTsR^46^. In ANTsPy, the function ants.registration(…) is used to register a pair of images or a pair of image sets where type_of_transform is a user-specified option that invokes a specific parameter set. For example type_of_transform=′antsRegistrationSyNQuick[s]′ encapsulates an oft-used parameter set for quick registration whereas type_of_transform=′antsRegistrationSyN[s]′ is a more aggressive alternative. Transforming images using the derived transforms is performed via the ants.apply_transforms(…) function.

Initially, linear optimization is initialized with center of (intensity) mass alignment typically followed by optimization of both rigid and affine transforms using the mutual information similarity metric. This is followed by diffeomorphic deformable alignment using symmetric normalization (SyN) with Gaussian^51^ or B-spline regularization^67^ where the forward transform is invertible and differentiable. The similarity metric employed at this latter stage is typically either neighborhood cross-correlation or mutual information. Note that these parameter sets are robust to input image type (e.g., light sheet fluorescence microscopy, Nissl staining, and the various MRI modalities) and are adaptable to mouse image geometry and scaling. Further details can be found in the various documentation sources for these ANTsX packages.

#### 4.1.3 Template generation

ANTsX provides functionality for constructing templates from a set (or multi-modal sets) of input images as originally described^59^ and recently used to create the DevCCF templates^16^. An initial template estimate is constructed from an existing subject image or a voxelwise average derived from a rigid pre-alignment of the image population. Pairwise registration between each subject and the current template estimate is performed using the Symmetric Normalization (SyN) algorithm^51^. The template estimate is updated by warping all subjects to the space of the template, performing a voxelwise average, and then performing a “shape update” of this latter image by warping it by the average inverse deformation, thus yielding a mean image of the population in terms of both intensity and shape. The corresponding ANTsPy function is ants.build_template(…).

#### 4.1.4 Visualization

To complement the well-known visualization capabilities of R and Python, e.g., ggplot2 and matplotlib, respectively, image-specific visualization capabilities are available in the ants.plot(…) function (Python). These are capable of illustrating multiple slices in different orientations with other image overlays and label images.

### 4.2 Mapping fMOST data to AllenCCFv3

#### 4.2.1 Preprocessing

- *Downsampling*. The first challenge when mapping fMOST images into the AllenCCFv3 is addressing the resolution scale of the data. Native fMOST data from an individual specimen can range in the order of terabytes, which leads to two main problems. First, volumetric registration methods (particularly those estimating local deformation) have high computational complexity and typically cannot operate on such high-resolution data under reasonable memory and runtime constraints. Second, the resolution of the AllenCCFv3 atlas is much lower than the fMOST data, thus the mapping process will cause much of the high-resolution information in the fMOST images to be lost regardless. Thus, we perform a cubic B-spline downsampling of the fMOST data to reduce the resolution of each image to match the isotropic 25 *μm* voxel resolution of the AllenCCFv3 intensity atlas using ants.resample_image(…). An important detail to note is that while the fMOST images and atlas are downsampled, the mapping learned during the registration is assumed to be continuous. Thus, after establishing the mapping to the AllenCCFv3, we can interpolate the learned mapping and apply it directly to the high-resolution native data directly to transform any spatially aligned data (such as the single-cell neuron reconstructions) into the AllenCCFv3.
- *Stripe artifact removal*. Repetitive pattern artifacts are a common challenge in fMOST imaging where inhomogeneity during the cutting and imaging of different sections can leave stripes of hyper- and hypo-intensity across the image. These stripe artifacts can be latched onto by the registration algorithm as unintended features that are then misregistered to non-analogous structures in the AllenCCFv3. We address these artifacts by fitting a 3-D bandstop (notch) filter to target the frequency of the stripe patterns and removing them prior to the image registration.
- *Inhomogeneity correction*. Regional intensity inhomogeneity can also occur within and between sections in fMOST imaging due to staining or lighting irregularity during acquisition. Similar to stripe artifacts, intensity gradients due to inhomogeneity can be misconstrued as features during the mapping and result in matching of non-corresponding structures. Our pipeline addresses these intensity inhomogeneities using N4 bias field correction^52^, ants.n4_bias_field_correction(…).

#### 4.2.2 Steps for spatial normalization to AllenCCFv3

1. *Average fMOST atlas as an intermediate target*. Due to the preparation of the mouse brain for fMOST imaging, the resulting structure in the mouse brain has several large morphological deviations from the AllenCCFv3 atlas. Most notable of these is an enlargement of the ventricles, and compression of cortical structures. In addition, there is poor intensity correspondence for the same anatomic features due to intensity dissimilarity between imaging modalities. We have found that standard intensity-base registration is insufficient to capture the significant deformations required to map these structures correctly into the AllenCCFv3. We address this challenge in ANTsX by using explicitly corresponding parcellations of the brain, ventricles and surrounding structures to directly recover these large morphological differences. However, generating these parcellations for each individual mouse brain is a labor-intensive task. Our solution is to create an average atlas whose mapping to AllenCCFv3 encapsulates these large morphological differences to serve as an intermediate registration point. This has the advantage of only needing to generate one set of corresponding annotations which is used to register between the two atlas spaces. New images are first aligned to the fMOST average atlas, which shares common intensity and morphological features and thus can be achieved through standard intensity-based registration.
2. *Average fMOST atlas construction*. An intensity and shape-based contralaterally symmetric average of the fMOST image data is constructed from 30 images and their contralateral flipped versions. We ran three iterations of the atlas construction using the default settings. Additional iterations (up to six) were evaluated and showed minimal changes to the final atlas construction, suggesting a convergence of the algorithm.
3. *fMOST atlas to AllenCCFv3 alignment*. Alignment between the fMOST average atlas and AllenCCFv3 was performed using a one-time annotation-driven approach. Label-to-label registration is used to align 7 corresponding annotations in both atlases in the following: 1) brain mask/ventricles, 2) caudate/putamen, 3) fimbria, 4) posterior choroid plexus, 5) optic chiasm, 6) anterior choroid plexus, and 7) habenular commissure. The alignments were performed sequentially, with the largest, most relevant structures being aligned first using coarse registration parameters, followed by other structures using finer parameters. This coarse-to-fine approach allows us to address large morphological differences (such as brain shape and ventricle expansion) at the start of registration and then progressively refine the mapping using the smaller structures. The overall ordering of these structures was determined manually by an expert anatomist, where anatomical misregistration after each step of the registration was evaluated and used to determine which structure should be used in the subsequent iteration to best improve the alignment. The transformation from this one-time expert-guided alignment is preserved and used as the canonical fMOST atlas to AllenCCFv3 mapping in the pipeline.
4. *Alignment of individual fMOST mouse brains*. The canonical transformation between the fMOST atlas and AllenCCFv3 greatly simplifies the registration of new individual fMOST mouse brains into the AllenCCFv3. Each new image is first registered into the fMOST average atlas, which shares intensity, modality, and morphological characteristics. This allows us to leverage standard, intensity-based registration functionality^86^ available in ANTsX to perform this alignment. Transformations are then concatenated to the original fMOST image to move it into the AllenCCFv3 space using ants.apply_transforms(…).
5. *Transformation of single cell neurons*. A key feature of fMOST imaging is the ability to reconstruct and examine whole-brain single neuron projections^79^. Spatial mapping of these neurons from individual brains into the AllenCCFv3 allows investigators to study different neuron types within the same space and characterize their morphology with respect to their transcriptomics. Mappings found between the fMOST image and the AllenCCFv3 using our pipeline can be applied in this way to fMOST neuron reconstruction point set data using ants.apply_transforms_to_points(..).

### 4.3 Mapping MERFISH data to AllenCCFv3

#### 4.3.1 Preprocessing

- *Initial volume reconstruction*. Alignment of MERFISH data into a 3-D atlas space requires an estimation of anatomical structure within the data. For each section, this anatomic reference image was created by aggregating the number of detected genetic markers (across all probes) within each pixel of a 10 × 10*μm*^2^ grid to match the resolution of the 10*μm* AllenCCFv3 atlas. These reference image sections are then coarsely reoriented and aligned across sections using manual annotations of the most dorsal and ventral points of the midline. The procedure produces an anatomic image stack that serves as an initialization for further global mappings into the AllenCCFv3.
- *Anatomical correspondence labeling*. Mapping the MERFISH data into the AllenCCFv3 requires us to establish correspondence between the anatomy depicted in the MERFISH and AllenCCFv3 data. Intensity-based features in MERFISH data are not sufficiently apparent to establish this correspondence, so we need to generate instead corresponding anatomical labelings of both images with which to drive registration. These labels are already available as part of the AllenCCFv3; thus, the main challenge is deriving analogous labels from the spatial transcriptomic maps of the MERFISH data. Toward this end, we assigned each cell from the scRNA-seq dataset to one of the following major regions: cerebellum, CTXsp, hindbrain, HPF, hypothalamus, isocortex, LSX, midbrain, OLF, PAL, sAMY, STRd, STRv, thalamus and hindbrain. A label map of each section was generated for each region by aggregating the cells assigned to that region within a 10 × 10*μm*^2^ grid. The same approach was used to generate more fine grained region specific landmarks (i.e., cortical layers, habenula, IC). Unlike the broad labels which cover large swaths of the section these regions are highly specific to certain parts of the section. Once cells in the MERFISH data are labeled, morphological dilation is used to provide full regional labels for alignment into the AllenCCFv3.
- *Section matching*. Since the MERFISH data is acquired as sections, its 3-D orientation may not be fully accounted for during the volume reconstruction step, due to the particular cutting angle. This can lead to obliqueness artifacts in the section where certain structures can appear to be larger or smaller, or missing outright from the section. To address this, we first use a global alignment to match the orientations of the MERFISH sections to the atlas space. In our pipeline, this section matching is performed in the reverse direction by performing a global affine transformation of the AllenCCFv3 into the MERFISH data space, and then resampling digital sections from the AllenCCFv3 to match each MERFISH section. This approach limits the overall transformation and thus resampling that is applied to the MERFISH data, and, since the AllenCCFv3 is densely sampled, it also reduces in-plane artifacts that result from missing sections or undefined spacing in the MERFISH data.

#### 4.3.2 2.5D deformable, landmark-driven alignment to AllenCCFv3

After global alignment of the AllenCCFv3 into the MERFISH dataset, 2D per-section deformable refinements are used to address local differences between the MERFISH sections and the resampled AllenCCFv3 sections. Nine registrations were performed in sequence using a single label at each iteration in the following order: 1) brain mask, 2) isocortex (layer 2+3), 3) isocortex (layer 5), 4) isocortex (layer 6), 5) striatum, 6) medial habenula, 7) lateral habenula, 8) thalamus, and 9) hippocampus. This ordering was determined empirically by an expert anatomist who prioritized which structure to use in each iteration by evaluating the anatomical alignment from the previous iteration. Global and local mappings are then all concatenated (with appropriate inversions) to create the final mapping between the MERFISH data and AllenCCFv3. This mapping is then used to provide a point-to-point correspondence between the original MERFISH coordinate space and the AllenCCFv3 space, thus allowing mapping of individual genes and cell types located in the MERFISH data to be directly mapped into the AllenCCFv3.

### 4.4 DevCCF velocity flow transformation model

Given multiple, linearly or non-linearly ordered point sets where individual points across the sets are in one-to-one correspondence, we developed an approach for generating a velocity flow transformation model to describe a time-varying diffeomorphic mapping as a variant of the landmark matching solution. Integration of the resulting velocity field can then be used to describe the displacement between any two time points within this time-parameterized domain. Regularization of the sparse correspondence between point sets is performed using a generalized B-spline scattered data approximation technique^85^, also created by the ANTsX developers and contributed to ITK.

#### 4.4.1 Velocity field optimization

To apply this methodology to the developmental templates^16^, we coalesced the manual annotations of the developmental templates into 26 common anatomical regions (see Figure 3). We then used these regions to generate invertible transformations between successive time points. Specifically each label was used to create a pair of single region images resulting in 26 pairs of “source” and “target” images. The multiple image pairs were simultaneously used to iteratively estimate a diffeomorphic pairwise transform. Given the seven atlases E11.5, E13.5, E15.5, E18.5, P4, P14, and P56, this resulted in 6 sets of transforms between successive time points. Approximately 10^6^ points were randomly sampled labelwise in the P56 template space and propagated to each successive atlas providing the point sets for constructing the velocity flow model. Approximately 125 iterations resulted in a steady convergence based on the average Euclidean norm between transformed point sets. Ten integration points were used and point sets were distributed along the temporal dimension using a log transform for a more evenly spaced sampling. For additional information a help menu is available for the ANTsPy function ants.fit_time_varying_transform_to_point_sets(…).

### 4.5 ANTsXNet mouse brain applications

#### 4.5.1 General notes regarding deep learning training

All network-based approaches described below were implemented and organized in the ANTsXNet libraries comprising Python (ANTsPyNet) and R (ANTsRNet) analogs using the Keras/Tensorflow libraries available as open-source in ANTsX GitHub repositories. For the various applications, both share the identically trained weights for mutual reproducibility. For all GPU training, we used Python scripts for creating custom batch generators which we maintain in a separate GitHub repository for public availability (https://github.com/ntustison/ANTsXNetTraining). These scripts provide details such as batch size, choice of loss function, and network parameters. In terms of GPU hardware, all training was done on a DGX (GPUs: 4X Tesla V100, system memory: 256 GB LRDIMM DDR4).

Data augmentation is crucial for generalizability and accuracy of the trained networks. Intensity-based data augmentation consisted of randomly added noise (i.e., Gaussian, shot, salt-and-pepper), simulated bias fields based on N4 bias field modeling, and histogram warping for mimicking well-known MRI intensity nonlinearities^46,88^. These augmentation techniques are available in ANTsXNet (only ANTsPyNet versions are listed with ANTsRNet versions available) and include:

- image noise: ants.add_noise_to_image(…),
- simulated bias field: antspynet.simulate_bias_field(…), and
- nonlinear intensity warping: antspynet.histogram_warp_image_intensities(…).

Shape-based data augmentation used both random linear and nonlinear deformations in addition to anisotropic resampling in the three canonical orientations to mimic frequently used acquisition protocols for mice brains:

- random spatial warping: antspynet.randomly_transform_image_data(…) and
- anisotropic resampling: ants.resample_image(…).

#### 4.5.2 Brain extraction

Similar to human neuroimage processing, brain extraction is a crucial preprocessing step for accurate brain mapping. We developed similar functionality for T2-weighted mouse brains. This network uses a conventional U-net architecture^89^ and, in ANTsPyNet, this functionality is available in the program antspynet.mouse_brain_extraction(…). For the two-shot T2-weighted brain extraction network, two brain templates were generated along with their masks. One of the templates was generated from orthogonal multi-plane, high resolution data^69^ which were combined to synthesize isotropic volumetric data using the B-spline fitting algorithm^85^. This algorithm is encapsulated in ants.fit_bspline_object_to_scattered_data(…) where the input is the set of voxel intensity values and each associated physical location. Since each point can be assigned a confidence weight, we use the normalized gradient value to more heavily weight edge regions. Although both template/mask pairs are available in the GitHub repository associated with this work, the synthesized volumetric B-spline T2-weighted pair is available within ANTsXNet through the calls:

- template: antspynet.get_antsxnet_data(″bsplineT2MouseTemplate″) and
- mask: antspynet.get_antsxnet_data(″bsplineT2MouseTemplateBrainMask″).

#### 4.5.3 Brain parcellation

The T2-weighted brain parcellation network is also based on a 3-D U-net architecture and the T2-w DevCCF P56 template component with extensive data augmentation, as described previously. Intensity differences between the template and any brain extracted input image are minimized through the use of the rank intensity transform (ants.rank_intensity(…)). Shape differences are reduced by the additional preprocessing step of warping the brain extracted input image to the template. Additional input channels include the prior probability images created from the template parcellation. These images are also available through the ANTsXNet get_antsxnet_data(…) interface.

## Figures and Tables

**Figure 2:**
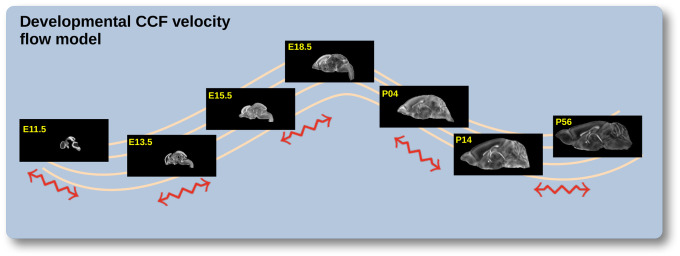
The spatial transformation between any two time points within the continuous DevCCF longitudinal developmental trajectory is available through the use of ANTsX functionality for generating a velocity flow model.

**Figure 9:**
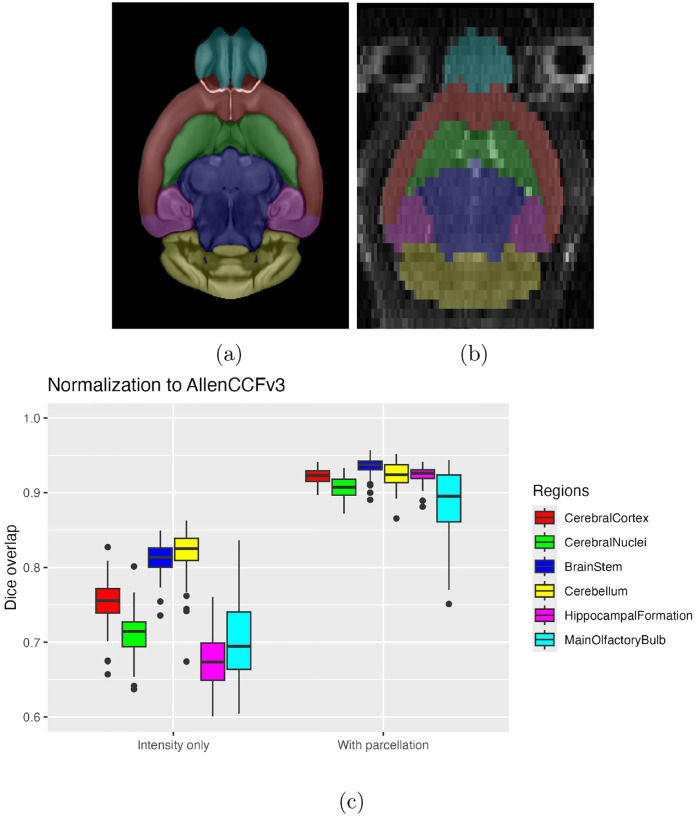
Evaluation of the ANTsX mouse brain parcellation on the same dataset. (a) T2-w DevCCF P56 with the described parcellation consisting of the cerebral cortex, nuclei, brain stem, cerebellum, main olfactory bulb, and hippocampal formation. (b) Sample subject (NR5 Day 0) with the proposed deep learning-based segmentation. (c) Dice overlap for comparing the regional alignments between registration using intensity information only and using intensity with the given parcellation scheme.

**Table 1:** Sampling of ANTsX functionality

*ANTsPy: Preprocessing*	
bias field correction	n4_bias_field_correction(…)
image denoising	denoise_image(…)
*ANTsPy: Registration*	
image registration	registration(…)
image transformation	apply_transforms(…)
template generation	build_template(…)
landmark registration	fit_transform_to_paired_points(…)
time-varying landmark reg.	fit_time_varying_transform_to_point_sets(…)
integrate velocity field	integrate_velocity_field(…)
invert displacement field	invert_displacement_field(…)
*ANTsPy: Segmentation*	
MRF-based segmentation	atropos(…)
Joint label fusion	joint_label_fusion(…)
diffeormorphic thickness	kelly_kapowski(…)
*ANTsPy: Miscellaneous*	
Regional intensity statistics	label_stats(…)
Regional shape measures	label_geometry_measures(…)
B-spline approximation	fit_bspline_object_to_scattered_data(…)
Visualize images and overlays	plot(…)
*ANTsPyNet: Mouse-specific*	
brain extraction	mouse_brain_extraction(…modality=“t2”…)
brain parcellation	mouse_brain_parcellation(…)
cortical thickness	mouse_cortical_thickness(…)
super resolution	mouse_histology_super_resolution(…)

ANTsX provides state-of-the-art functionality for processing biomedical image data. Such tools, including deep learning networks, support a variety of mapping-related tasks. A more comprehensive listing of ANTsX tools with self-contained R and Python examples is provided as a gist page on GitHub (https://tinyurl.com/antsxtutorial).

## Data Availability

All data and software used in this work are publicly available. The DevCCF atlas is available at https://kimlab.io/brain-map/DevCCF/. ANTsPy, ANTsR, ANTsPyNet, and ANTsRNet are available through GitHub at the ANTsX Ecosystem (https://github.com/ANTsX). Training scripts for all deep learning functionality in ANTsXNet can also be found on GitHub (https://github.com/ntustison/ANTsXNetTraining). A GitHub repository specifically pertaining to the AllenCCFv3 mapping is available at https://github.com/dontminchenit/CCFAlignmentToolkit. For the other two contributions contained in this work, the longitudinal DevCCF mapping and mouse cortical thickness pipeline, we refer the interested reader to https://github.com/ntustison/ANTsXMouseBrainMapping.
